# Expression and function analysis of CRABP2 and FABP5, and their ratio in esophageal squamous cell carcinoma

**DOI:** 10.1515/med-2021-0350

**Published:** 2021-09-27

**Authors:** Mengyan Li, Chao Li, Pengfei Lu, Bo Wang, Yongmei Gao, Wengying Liu, Yan Shi, Yuqing Ma

**Affiliations:** Departments of Pathology, The First Affiliated Hospital, Xinjiang Medical University, 393 Liyushan Road, Urumqi, Xinjiang 830011, People’s Republic of China; Departments of Pathology, The First Affiliated Hospital, Xinjiang Medical University, Urumqi, Xinjiang 830011, People’s Republic of China; Departments of Oncology, The First Affiliated Hospital, Xinjiang Medical University, Urumqi, Xinjiang 830011, People’s Republic of China

**Keywords:** esophageal squamous cell carcinoma, public database, immunohistochemistry, CRABP2, FABP5

## Abstract

**Objective:**

The purpose of this study was to explore the effect of CRABP2 and FABP5, and their ratio on prognosis in esophageal squamous cell carcinoma.

**Methods:**

The expression data of CRABP2 in esophageal cancer in TCGA and GEO were collected by the public database GEPIA. The expression levels of CRABP2 and FABP5 were examined using immunohistochemistry. The relationship between the two proteins and related clinicopathological parameters were analyzed by *χ* ^2^ test. Survival analysis was used to investigate the effect of CRABP2 and FABP5, and their ratio on prognosis.

**Results:**

Compared with normal esophageal mucosal epithelium, there was lower CRABP2 gene mRNA in the esophageal cancer tissue, and the difference was statistically significant (*p* < 0.01). For the expression level, no significant difference was observed in patients with stages I–IV in esophageal cancer. Immunohistochemistry showed that CRABP2 and FABP5 were both highly expressed in normal esophageal squamous epithelial cells at 100 and 94.1%, while lower in ESCC (75.6 and 58.7%). There was a significant difference in the expression between cancer and adjacent tissues (*p* < 0.001). No inherent relationship was manifested between the CRABP2 expression and the clinical parameters of the ESCC. The expression of FABP5 was related to lymph node metastasis (*p* = 0.032), the depth of invasion (*p* = 0.041), and the AJCC stage (*p* = 0.013). The ratio of CRABP2 and FABP5 was related to ethnicity (*p* = 0.001), nerve invasion (*p* = 0.031), and postoperative treatment (*p* = 0.038). CRABP2 is positively associated with FABP5 (*r* = 0.156, *p* = 0.041) and the ratio (*r* = 0.334, *p* = 0.000), while there was a negative correlation between FABP5 and the ratio (*r* = −0.269, *p* = 0.000). Patients with CRABP2-positive expression had a significantly longer overall survival than patients with CRABP2-negative expression (*p* = 0.025).

**Conclusion:**

CRABP2 as a suppressor factor is expected to be a potential prognosis marker for esophageal squamous cell carcinoma.

## 1 Introduction

Esophageal cancer (EC) is a malignant tumor originating in the esophageal epithelium. In the global cancer statistics of 2018, EC was seventh in morbidity and sixth in the mortality rate, with a morbidity rate of 3.2% and a mortality rate of 5.3% [1]. In China, esophageal squamous cell carcinoma (ESCC) accounts for the vast majority and the incidence of ESCC ranks sixth [2], with the situation being not optimistic. The exact cause of esophageal cancer is unknown. Esophagectomy remains the primary treatment for esophageal cancer. In recent years, many differential gene expression changes in esophageal cancer are related to the pathological mechanism, treatment, and prognosis of the disease; however, the treatment and prognosis improvement of esophageal cancer for known targets like EGFR and HER-2 are still insufficient.

Cellular retinoic acid-binding protein 2 (CRABP2), mainly found in the skin, uterus, ovary, and nerve choroid plexus, is a small molecule cytoplasmic shuttle protein containing 138 amino acid residues, which promotes retinoic acid (RA) binding to its cognate receptor complex and transfers to the nucleus [3,4]. Some scholars believe that CRABP2 is an oncogene while others declare that CRABP2 is a tumor suppressor gene. Although it has been reported that CRABP2 is low expression in ESCC, the relationship among CRABP2, prognosis, and interaction protein is still unclear.

Fatty-acid-binding protein 5 (FABP5) is a highly conserved cytoplasmic protein that binds to long-chain fatty acids and other hydrophobic ligands [5]. Like CRABP2, it is an intracellular lipid-binding protein (iLBP) member of the family, The two proteins play important roles in the retinoic acid signaling pathway.

Retinoic acid (RA) is a metabolite of vitamin A. The latest research revealed that there was a close relationship between the metabolism of retinoic acid and the occurrence of esophageal cancer in the Chinese population [6]. RA can participate in cell cycle arrest, apoptosis, and differentiation through CRABP2; it also can affect cell survival, cell proliferation, and angiogenesis through FABP5 [7], the activation pathway seems to depend on the ratio of FABP5/CRABP2.

Gene chip technology and bioinformatics analysis methods have been widely used in the research of various diseases, especially in tumors. Bioinformatics methods such as screening differentially expressed genes (DEGs) and their functional enrichment analysis can help reveal the pathological mechanism of ESCC from the genetic level and contribute to clinical practice. In the present study, bioinformatics methods were used for data mining on gene chip databases, followed by immunohistochemistry in combination with clinical cases, making a preliminary study of the differential genes between ESCC and normal tissues.

Although it has been reported that the expression of CRABP2 is lower in ESCC, the prognostic value of CRABP2 and its relationship with the expression of competitive protein FABP5 is uncertain, as well as the relationship with the ratio of CRABP2 and FABP5 and prognosis.

Through clarifying the expression and clinical significance of CRABP2 in ESCC, we aim to explore the biological function of CRABP2, the relationship between CRABP2 and its closely related proteins FABP5, and provide new clues for molecular targeted therapy of ESCC.

## 2 Methods

### 2.1 Expression of CRABP2 in esophageal cancer and normal esophageal tissues

The basic clinical information of tumor cases, such as demographic data, treatment process, clinical stage, tumor pathology, survival status, and any other related information (mRNA, microRNA, copy number, mutation, protein, methylation information, etc.) was collected from TCGA (The Cancer Genome Atlas http://cancergenome.nih.gov/) and GEO (https://www.ncbi.nlm.nih.gov/gds/) database. According to TGGA, the expression of CRABP2 mRNA in EC tissues and normal tissues was analyzed using GEPIA.

### 2.2 The expression status of different stages of EC patients

The expression of the CRABP2 gene in different stages of EC was evaluated by the GEPIA database.

### 2.3 Survival analysis

The relationship between the CRABP2 gene expression level and the prognosis of patients with EC was analyzed by the GEPIA database.

### 2.4 Protein interaction network

Related genes and a scatter diagram were derived from the GEPIA database. The CRABP2 gene analysis of protein–protein, genetic interactions, pathways, and protein co-expression networks were dependent on the Genemania database.

### 2.5 Gene function

WebGestalt is an online website for gene function enrichment analysis. To explore the involved cell components, biological processes, and biological functions, the key genes interacting with CRABP2 were analyzed by WebGestalt.

### 2.6 Tissue microarray (TMA)

TMAs were constructed by the Department of Pathology, The First Affiliated Hospital of Xinjiang Medical University, and technical supports were provided by Shanghai Outdo Biotech Company (Shanghai, China). The TMA contained a total of 240 ESCC patients and 240 matched normal tissues. Paraffin-embedded tissue in each core was obtained from the non-necrotic area of the carcinoma and/or noncarcinoma foci.

### 2.7 Patients

This study included a retrospective series of 240 patients with ESCC and treated at the First Affiliated Hospital of Xinjiang Medical University from January 2008 to June 2019. The diagnoses of all cases were confirmed by an expert pathology review (SF). The inclusion criteria were as follows: (1) patients with ESCC; (2) patients who did not receive radiotherapy or chemotherapy before surgery; (3) the esophagus was the primary lesion site; and (4) patients of Han or Kazakh ethnicity. The exclusion criteria included the following: (1) patients with adenocarcinoma of the esophagus; (2) patients received radiotherapy or chemotherapy before surgery; (3) patients with tumor metastasis to the esophagus; (4) other ethnic groups, including Uyghurs, Mongolians, etc.; (5) patients who died during the operation and in hospital; and (6) patients with distant metastasis before operation.

According to the above criteria, 172 cases of esophageal squamous cell carcinoma (including 101 cases of Han nationality and 71 cases of Hazak nationality) were randomly collected, including their clinicopathological data: age (<60, ≥60), gender (male, female), tumor location (upper, middle, lower), tumor size (<3 cm, ≥3 cm), differentiation degree (well, moderate, poor), lymph node metastasis (yes, no), vascular invasion (yes, no), nerve invasion (yes, no), hematogenous metastasis (yes, no), AJCC (according to the eighth version of esophageal cancer released by American Joint Committee on Cancer [AJCC] and Union for International Cancer Control [UICC] in 2017, I + II, III + IV), depth of invasion (Mucosa, Muscularis, Full thickness), and postoperative treatment (chemotherapy/radiotherapy, no) (Table 1). The patients were clearly informed about the operation, material extraction, pathological examination, and experimental purpose.

**Table 1:** General characteristics of ESCC patients

Characteristics	*n* = 172	Percentage (%)
Age (years)		
>60	114	66.3
≤60	58	33.7
Tumor size (cm)		
<3	56	32.6
≥3	116	67.4
Gender		
Male	119	69.2
Female	53	30.8
Ethnicity		
Han	101	58.7
Kazakh	71	41.3
Degree of differentiation		
Well	33	19.2
Moderate	104	60.5
Poor	35	20.3
AJCC stage		
I + II	102	59.3
III + IV	70	40.7
Lymph node metastasis		
No	115	66.9
Yes	57	33.1
Tumor location		
Upper	9	5.2
Middle	108	62.8
Lower	55	32.0
Invasion depth		
Mucosa	10	5.8
Muscularis	63	36.6
Full thickness	99	57.6
Vascular invasion		
No	139	80.8
Yes	33	19.2
Nerve invasion		
No	137	79.7
Yes	35	20.3
Hematogenous metastasis		
No	150	87.2
Yes	22	12.8
Postoperative treatment		
No	103	59.9
Yes	69	40.1
CRABP2		
Low expression	42	24.4
High expression	130	75.6
FABP5		
Low expression	71	41.3
High expression	101	58.7
CRABP2/FABP5		
≥1	50	29.1
＜1	122	70.9

All the selected cases were patients with immersive esophageal squamous cell carcinoma without high-grade intraepithelial neoplasia and carcinoma *in situ*. According to 2020 CSCO esophagus cancer diagnosis and treatment guidelines, endoscopic mucosal resection can be considered for T1a tumors (involving mucosa but not submucosa), while esophagectomy can be used for T1b or deeper tumors. There was no T1a in the 172 patients and they were all T1b–T4. Radical resection of esophageal carcinoma and lymph node dissection was performed in all the selected patients, followed by esophageal reconstruction with gastroesophageal anastomosis.

It took about 4 months including experiment design, reagents purchase, data organization, regular follow-up, pre-experiment, formal experiment, result interpretation, statistical analysis, and completion of the first draft (from May 2020 to the end of August). Our follow-up time ended in July 2020 through inquiring about the medical records and telephone calls. Overall survival (OS) was calculated as the time interval between the date of surgery and death or last follow-up. Progression-free survival (PFS) was determined from the date of surgery to tumor recurrence or the last follow-up. The time range of OS and PFS is 1–108. The median follow-up time of OS was 28 months while the median follow-up time of PFS was 20 months.

**Ethics approval and consent to participate:** This study was approved by the ethics committee of the First Affiliated Hospital of Xinjiang Medical University. A Signed written informed consent was obtained from all participants before the study.**Patient consent for publication:** Not applicable.

### 2.8 Immunohistochemistry

Rabbit monoclonal anti-CRABP2 antibody (ab211927, Abcam, dilution, 1:2,000), rabbit monoclonal anti-FABP5 antibody (ab255276, Abcam, dilution,1:5,000) were purchased for the immunohistochemistry test.

Deparaffinization/hydration of TMAs was performed as follows: sliced specimens were incubated at 58–65°C for 1 h, On the day before the experiment, tissue chips were put into the 37°C oven overnight, softening the wax layer that covered the tissue chip. After dewaxing and dehydration, the tissue chip was put in a boiling EDTA repair solution (pH 9.0) and boiled for 15 min; After 30 min of cooling at room temperature, endogenous peroxidase was added and incubated for 20 min at room temperature. After rinsing with phosphate-buffered saline three times, the CRABP2 and FABP5 antibodies were put in the 37°C oven for 1 h. After cleaning, the goat antirabbit secondary antibody (PV-6001) was placed in a 37°C oven for 30 min. Finally, they were DAB stained solution to brown. Each experiment included a positive control slide. For negative control, the primary antibody was replaced with PBS. Both were located in the cytoplasm or nucleus.

The microscopic examination of each point of the tissue microarray was performed at the same incident light intensity and compensation intensity. Five high-power fields (×400) were selected at random. The evaluation was based on a semi-quantitative method described as follows: Staining index = percentage of positive cells × staining strength; the rule of staining intensity score: 0 point (Negative), 1 point (Light brown), 2 points (Brown), and 3 points (Dark brown). The rule of stained positive cells scores: 0 points (0–10%), 1 point (11–25%), 2 points (26–50%), 3 points (51–75%), and 4 points (76–100%). Above all, the staining index calculations were 0, 1, 2, 3, 4, 6, 8, 9, 12, respectively. For CRABP2, we define it as high expression if the total score is >1 point; otherwise, we define it as low expression. For FABP5, we define it as high expression if the total score is >6 points; otherwise, we define it as low expression. The total score of the tissue chip was independently completed by two pathologists who had no knowledge of the patient’s clinical case data. All scoring differences were resolved through discussion.

### 2.9 Statistical methods

Statistical analyses were performed by SPSS 26.0 (SPSS Inc, Chicago, IL, USA). The characteristics of the ESCC patients were compared using the *χ* ^2^ test. OS and progression-free survival (PFS) were assessed using the Kaplan–Meier method and the log-rank test. Multivariate analysis was carried out using the Cox proportional hazard regression model. *p* < 0.05 was considered statistically significant (*χ* ^2^ test was used for univariate analysis, *p* < 0.15).

## 3 Results

### 3.1 Results of differential expression of CRABP2 genes in ESCC and normal tissues in the database

**Figure 1:**
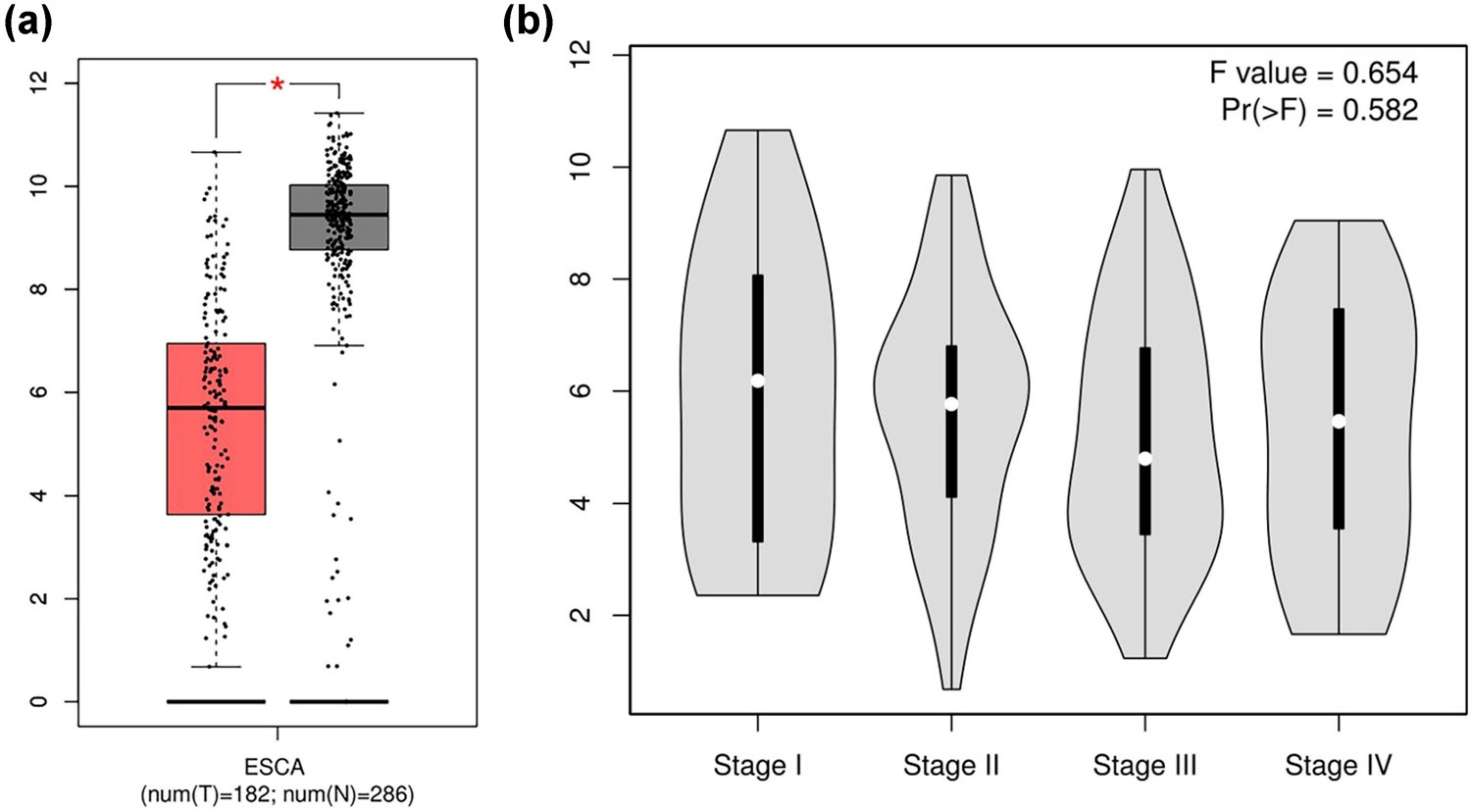
(a) Analysis of the expression level of CRABP2mRNA in esophageal cancer tissues and normal esophageal tissues based on the GEPIA database (red indicates esophageal cancer tissues, gray indicates normal esophageal tissues, and the difference is statistically significant). (b) The relationship between CRABP2 and tumor staging.

Through the online analysis of the GEPIA website, in Figure 1a, we compared the expression levels of CRABP2 mRNA in 286 cases of EC and 182 cases of esophageal normal tissues. It was found that the expression level in esophageal normal tissues was significantly higher than that in ESCC (*p* < 0.01).

Based on the analysis of the GEPIA database, Figure 1b shows that the expression level of CRABP2 in EC tissues with stages Ⅰ–IV was not statistically significant (*p* > 0.05).

**Figure 2:**
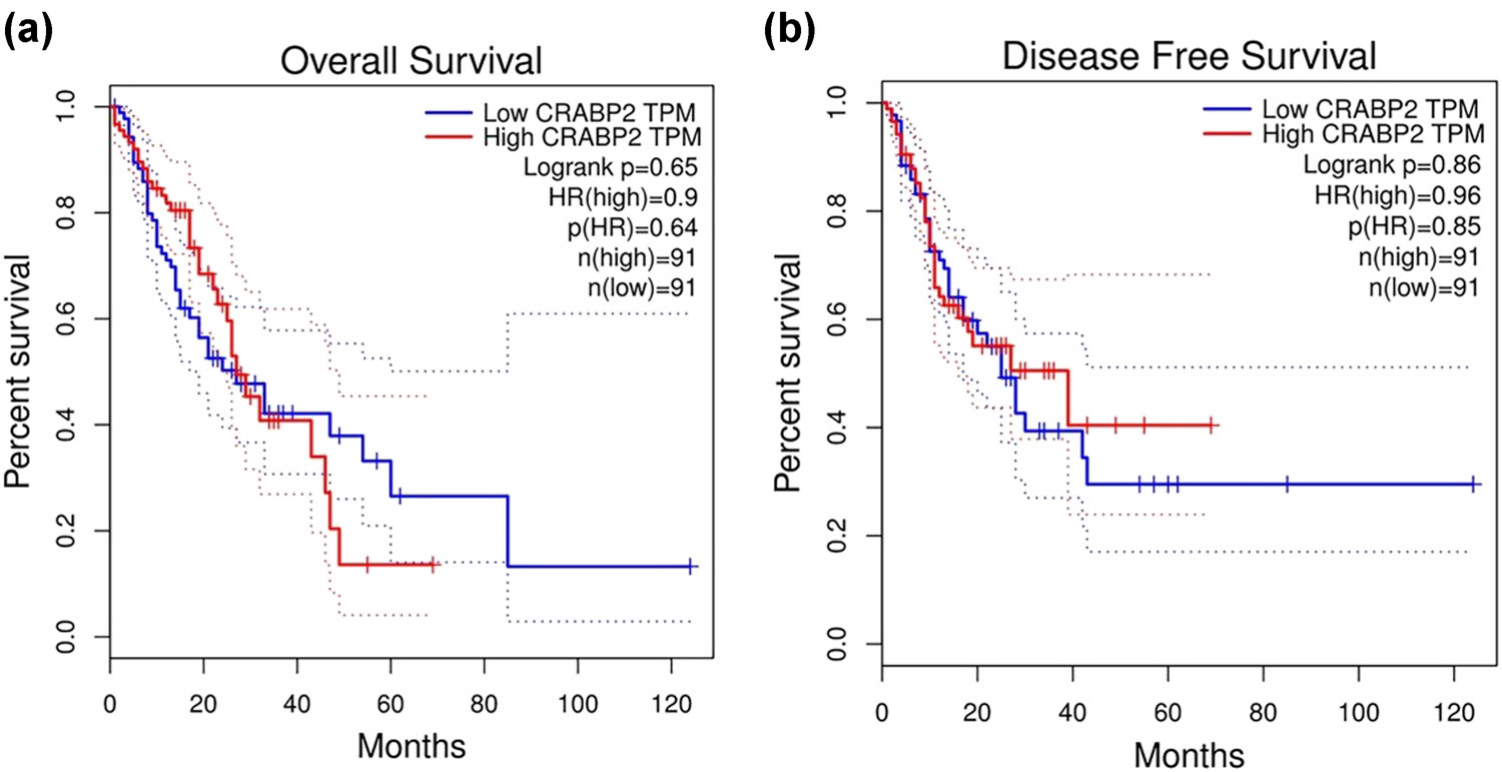
(a) Analysis of the overall survival of patients with esophageal cancer with different expression levels of CRABP2 in the GEO database. (b) Analysis of the disease free survival of patients with esophageal cancer with different expression levels of CRABP2 in the GEO database.

We used the GEPIA database to analyze the correlation between CRABP2 different expression levels and the OS and PFS of 182 EC patients. The patients were divided into the CRABP2 high expression group (*n* = 91) and the CRABP2 low expression group (*n* = 91) according to the median expression. The results were not statistically significant (Figure 2a and b).

### 3.2 Prediction and functional analysis of the CRABP2 protein interaction network and GO analysis

**Figure 3:**
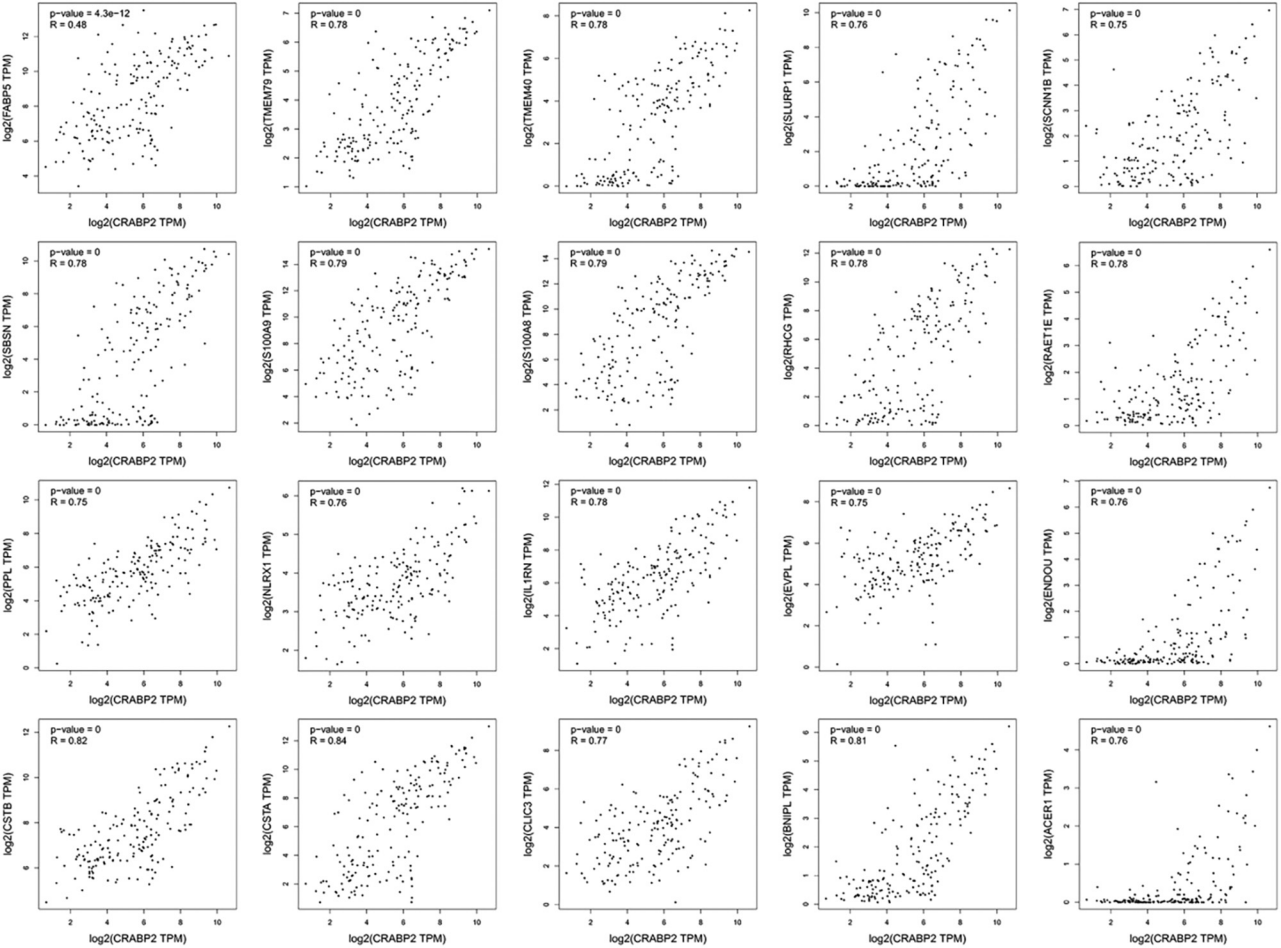
Correlation analysis between CRABP2 and each gene.

**Figure 4:**
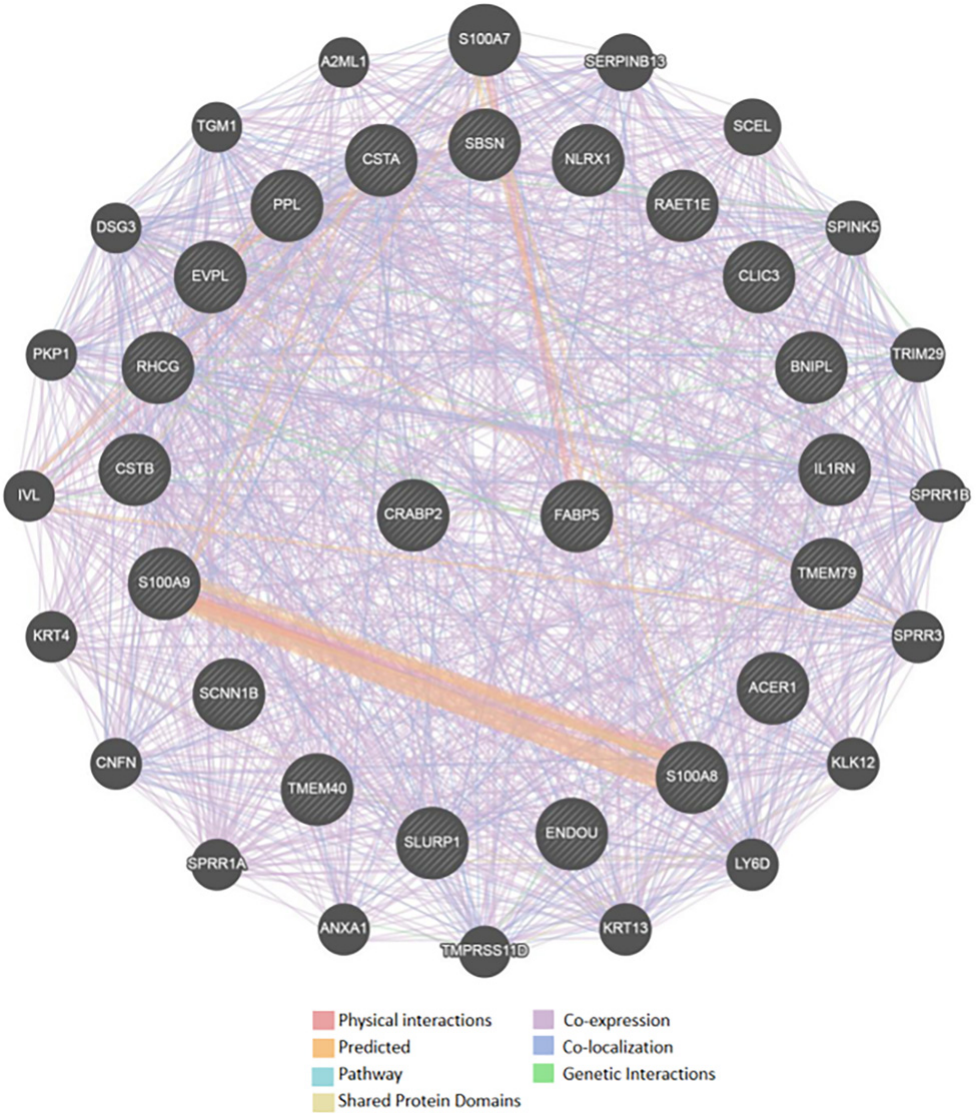
Genemania analysis of CRABP2 interaction proteins.

Using Genemania analysis, we screened a total of 20 CRABP2 interacting proteins: FABP5, CSTA, CSTB, BNIPL, S100A9, S100A8, SBSN, IL1RN, TMEM40, TMEM79, RHCG, RAET1E, CLIC3, ACER1, SLURP1, ENDOU, NLRX1, EVPL, SCNN1B, and PPL, and the interaction relationship included physical interaction, co-expression, sharing protein domains, and co-localization. The scatter plot of the correlation between CRABP2 and each gene is shown in Figure 3. The interactive network is shown in Figure 4.

**Figure 5:**
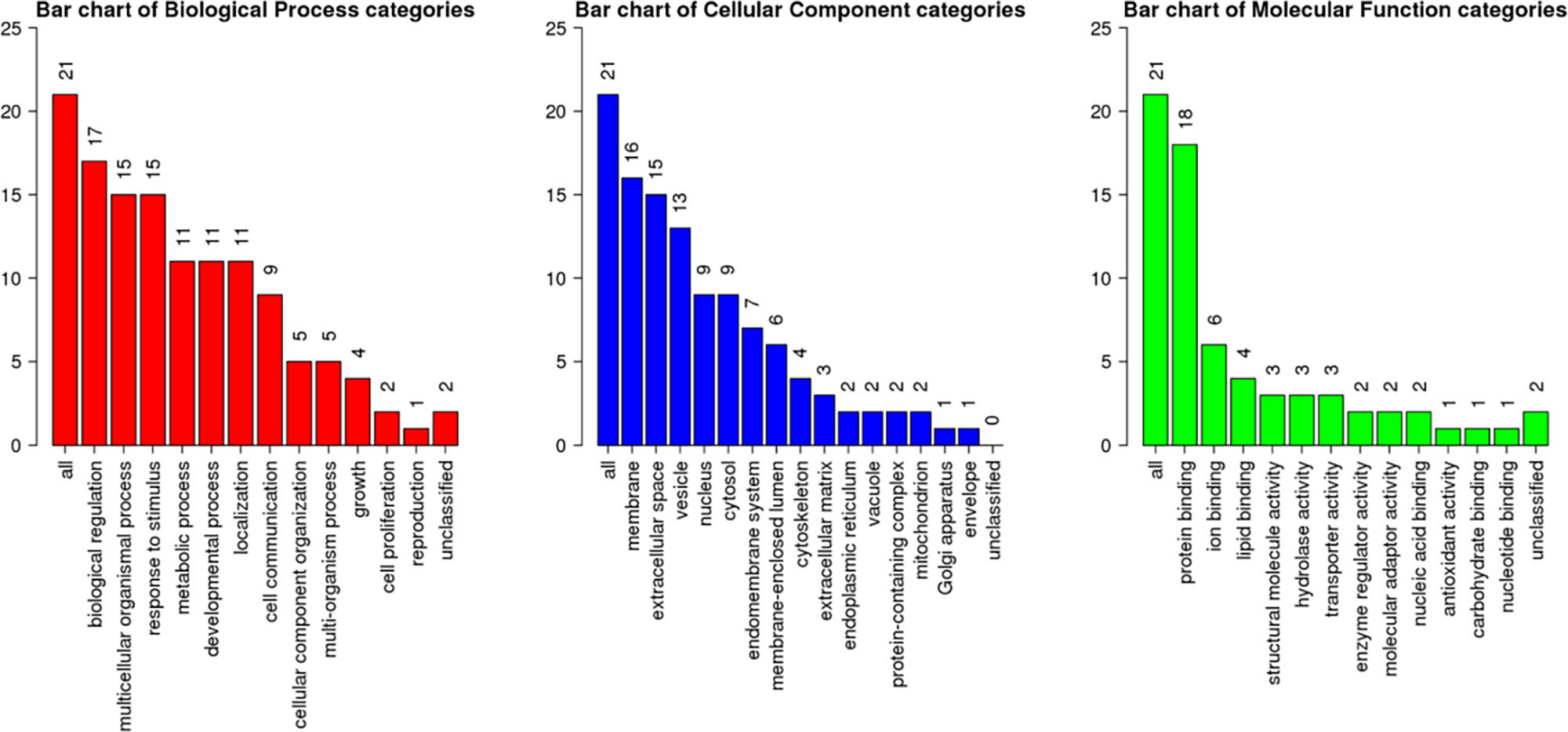
Analysis of biological processes, cell components, and molecular functions involved in the interaction with CRABP2 protein.

It is found that these proteins are mainly involved in biological regulation, multicellular biological processes, and responses to stimuli. CRABP2-related genes are located in a variety of cell components, including cell membrane, extracellular, cell vesicles, and so on. Molecular functions include binding proteins, participating in iron ion binding, etc. (Figure 5).

### 3.3 Clinicopathologic characteristics

The demographic data of the 172 patients with ESCC included in the study and the pathological characteristics are summarized in Table 1. The median age of patients at the time of diagnosis was 63 years (35–83 years). The patients were followed up for a mean of 28 months (range 1–108), and 130 (75.5%) patients died during the follow-up period.

### 3.4 Expression of CRABP2 and FABP5 in ESCC and normal esophageal tissues

**Figure 6:**
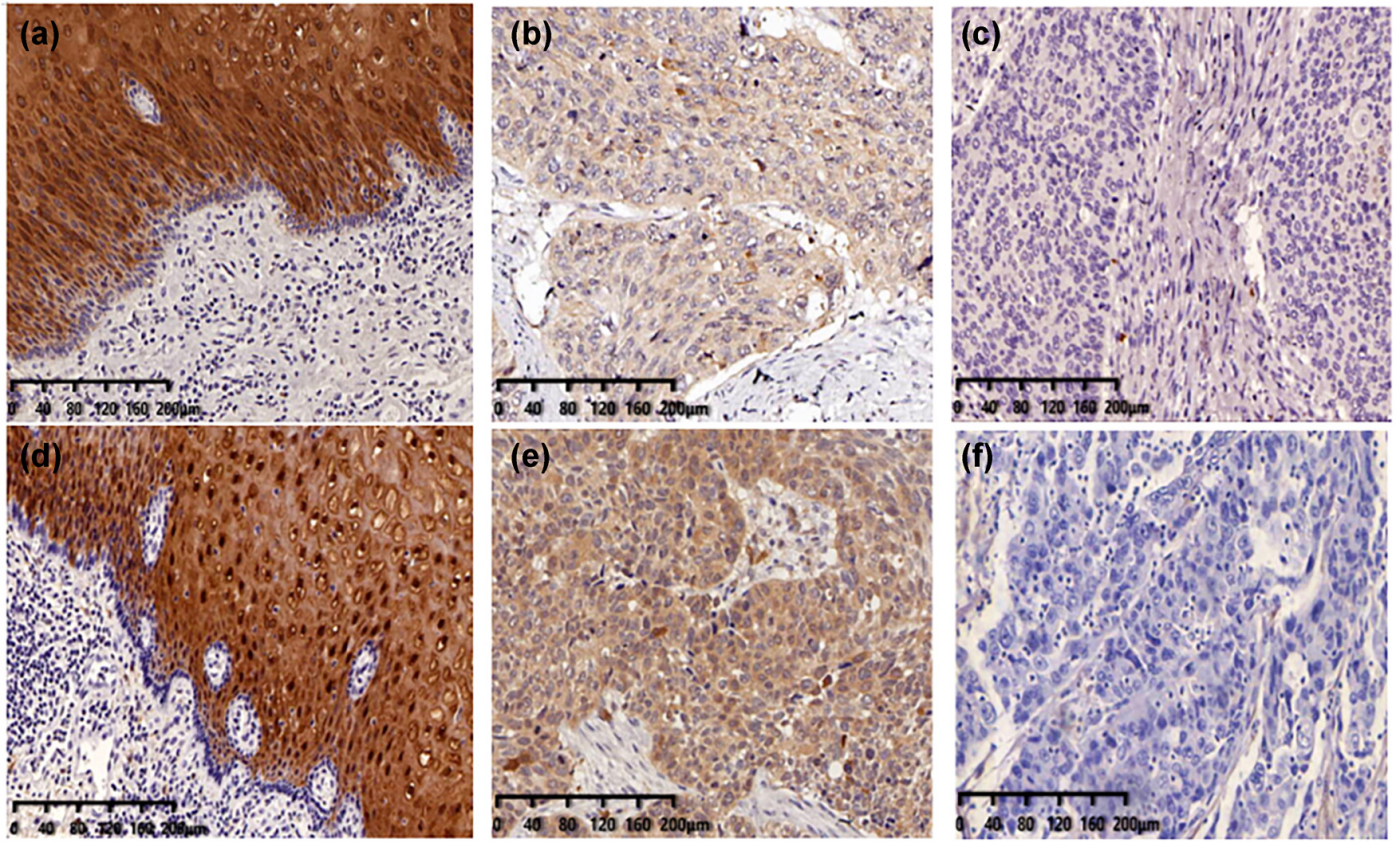
Immunohistochemical detection of the expression of CRABP2 and FABP5 in esophageal cancer and normal esophageal tissues. (a and b) CRABP2 is highly expressed in the normal esophageal squamous epithelium and esophageal squamous cell carcinoma; (c) low expression of CRABP2 in esophageal squamous cell carcinoma; (d and e) FABP5 is highly expressed in the normal esophageal squamous epithelium and esophageal squamous cell carcinoma; and (f) low expression of FABP5 in esophageal squamous cell carcinoma.

We detected CRABP2 and FABP5 in both adjacent normal esophageal mucosa epithelium and ESCC tissues by IHC. The representative IHC images of CRABP2 and FABP5 are presented in Figure 6.

CRABP2 was localized in cell nuclei and cytoplasm of ESCC and normal esophageal mucosa. Among 172 cases of the normal esophageal mucosa, CRABP2 was high in all patients. The high expression rate of CRABP2 in the normal esophageal mucosa was 100% (172/172). For the cancer specimens, CRABP2 high expression was observed in 75.6% (130/172) and the difference was statistically significant (Table 2). Moreover, the degree of expression was different, and the staining and scoring of CRABP2 in normal tissues of the esophagus were stronger, while those in ESCC were weaker. This proves that CRABP2 is a downregulated protein.

**Table 2:** Expression of CRABP2 and FABP5 in ESCC and adjacent tissues

	CRABP2			
	Low expression	High expression	Sum	*p*
Cancer	42	130	172	0.000
Control	0	172	172	
Sum	42	202	344	

		FABP5			
		Low expression	High expression	Sum	*p*
Cancer		71	101	172	<0.001
Control		10	162	172	
Sum		82	262	344	

FABP5 was localized in cell nuclei and cytoplasm of ESCC and normal esophageal mucosa. Among 172 cases of the normal esophageal mucosa, 162 were high expression and 10 were low expression. The high expression rate of FABP5 in normal esophageal mucosa was 94.1% (162/172). For the cancer specimens, FABP5 high expression was observed in 58.7% (101/172) and the difference was statistically significant (Table 2). The staining and scoring of FABP5 in normal tissues of the esophagus were stronger, while those in ESCC were weaker. FABP5 is a downregulated protein in cancer tissue, too.

### 3.5 Analysis of association between the expression of CRABP2 and FABP5 proteins and clinicopathological characteristics in ESCC

Table 3 reveals that the expression of CRABP2 was not associated with age, gender, degree of differentiation, invasion depth, vascular invasion, nerve invasion, hematogenous metastasis, and postoperative treatment in ESCC (*p* > 0.05).

**Table 3:** CRABP2, FABP5, CRABP2/FABP5 levels in esophageal squamous cell carcinoma and their relationship with clinicopathological characteristics

Items	CRABP2		*p*	FABP5		*p*	Ratio		*p*
	Low expression	High expression		Low expression	High expression		≥1	＜1	
Gender									
Male	30	89	0.717	51	68	0.529	82	37	0.381
Female	12	41		20	33		40	13	
Age									
≤60	16	42	0.490	20	38	0.197	42	16	0.760
>60	26	88		51	63		80	34	
Ethnicity									
Han	21	80	0.187	42	59	0.923	62	39	0.001
Kazakh	21	50		29	42		60	11	
Tumor location									
Upper	1	8	0.447	2	7	0.323	7	2	0.860
Middle	25	83		43	65		77	31	
Lower	16	39		26	29		38	17	
Tumor size									
<3 cm	9	47	0.077	27	29	0.06	35	21	0.091
≥3 cm	33	83		44	72		87	29	
Degree of differentiation									
Poor	12	23	0.102	19	16	0.180	21	14	0.214
Moderate	26	78		38	66		75	29	
Well	4	29		14	19		26	7	
Lymph node metastasis									
No	23	92	0.055	54	61	0.032	80	35	0.575
Yes	19	38		17	40		42	115	
Invasion depth									
Mucosa	1	9	0.421	6	4	0.041	6	4	0.142
Muscularis	14	49		32	31		40	23	
Full thickness	27	72		33	66		76	23	
AJCC stage									
I + II	21	81	0.158	50	52	0.013	70	32	0.422
III + IV	21	49		21	49		52	18	
Vascular invasion									
No	33	106	0.671	61	78	0.154	100	39	0.549
Yes	9	24		10	23		22	11	
Nerve invasion									
No	35	102	0.495	55	82	0.550	92	45	0.031
Yes	7	28		16	19		30	5	
Hematogenous metastasis									
No	38	112	0.466	63	87	0.616	105	45	0.483
Yes	4	18		8	14		17	5	
Postoperative treatment									
No	24	79	0.677	47	56	0.157	67	36	0.038
Chemotherapy/radiotherapy	18	51		24	45		55	14	

The expression of FABP5 was correlated with the lymph node metastasis (*p* = 0.032), invasion depth (*p* = 0.041), and AJCC stage (*p* = 0.013), while no association was seen with age, gender, ethnicity, vascular invasion, nerve invasion, hematogenous metastasis, and postoperative treatment in ESCC (*p* > 0.05).

We also analyzed the relationship between the CRABP2 and FABP5 ratio and clinical parameters. The ratio was correlated with ethnicity (*p* = 0.001), nerve invasion (*p* = 0.031), and postoperative treatment (*p* = 0.038), while no association was seen with age, gender, tumor location, tumor size, invasion depth, AJCC, lymph node metastasis, vascular invasion, and hematogenous metastasis in ESCC (*p* > 0.05).

### 3.6 The relationship between CRABP2 and FABP5

The pairwise correlation among CRABP2, FABP5, and the ratio of the two proteins was evaluated by the Spearman rank correlation test.Table 4 shows that CRABP2 had a positive correlation with FABP5 (*r* = 0.156, *p* = 0.041). There was an positive correlation between the expression of CRABP2 and the ratio (*r* = 0.334, *p* = 0.000). Meanwhile, FABP5 had a negative correlation with the ratio (*r* = −0.269, *p* = 0.000). The above data suggest a correlation between CRABP2 and FABP5. Moreover, the result is similar to Genemania analysis, that is CRABP2 expression was positively correlated with FABP5 (*p* = 4.3 ×10^−12^, *r* = 0.48). CRABP2 and FABP5 may play different roles in ESCC.

**Table 4:** Pairwise association between CRABP2 and FABP5 proteins and their ratio

Parameter		CRABP2/FABP5	CRABP2
FABP5	*r*	−0.269	0.156
	*p*	0.000	0.041
CRABP2	*r*	0.334	—
	*p*	0.000	—

In conclusion, we conclude that CRABP2 and FABP5 are low expressed in ESCC, and CRABP2 expression is positively correlated with FABP5 expression. The expression and relationship between the two proteins may be related to tumor progression in ESCC.

### 3.7 Association clinical features in ESCC with patient survival

In univariate analysis, death and recurrence were regarded as dependent variables, and 16 factors were regared as arguments, such as the gender, ethnicity, degree of differentiation, depth of invasion, AJCC stage, lymph node metastasis, nerve invasion, hematogenous metastasis, CRABP2 expression, etc. In order to avoid missing some important factors, *p* = 0.15 was setted in univariate analysis. The result showed that the gender (*p* = 0.119), ethnicity (*p* = 0.118), differentiation of degree (*p* = 0.000), depth of invasion (*p* = 0.117), AJCC stage (*p* = 0.028), lymph node metastasis (*p* = 0.140), hematogenous metastasis (*p* = 0.020), nerve invasion (*p* = 0.045), and CRABP2 expression (*p* = 0.079) were the risk factors for OS of patients with ESCC (Table 5). Additionally, the differentiation of degree (*p* = 0.018), AJCC stage (*p* = 0.013), lymph node metastasis (*p* = 0.067), hematogenous metastasis (*p* = 0.094), and CRABP2 expression (*p* = 0.152) were the risk factors for PFS of patients with ESCC (Table 5).

**Table 5:** Univariate analysis of factors associated with overall survival and progression-free survival in ESCC patients

Characteristic	OS		PFS	
	*χ* ^2^	*p*-value	*χ* ^2^	*p*-value
Gender (female vs male)	2.433	0.119	3.207	0.201
Age (≤60 vs >60)	0.191	0.662	2.044	0.360
Ethnicity (Han vs Kazakh)	2.445	0.118	3.195	0.202
Tumor location (Up vs M vs L)	2.981	0.225	4.964	0.291
Tumor size (<3 vs ≥3 cm)	0.776	0.378	2.434	0.296
Degree of differentiation (PD vs MD vs WD)	17.092	0.000	11.872	0.018
Invasion depth (MA vs MS vs FT)	4.284	0.117	6.058	0.195
AJCC stage (I + II vs II + IV)	4.846	0.028	8.693	0.013
Lymph node metastasis (No vs YES)	2.183	0.140	5.402	0.067
Vascular invasion (No vs Yes)	0.861	0.354	0.627	0.731
Nerve invasion (No vs Yes)	4.018	0.045	3.236	0.198
Hematogenous metastasis (No vs Yes)	5.398	0.020	4.735	0.094
Postoperative treatment (No vs Che + Ra)	0.094	0.759	2.129	0.345
CRABP2 expression (high expression vs Low expression)	3.091	0.079	3.767	0.152
FABP5 expression (high expression vs low expression)	0.057	0.811	1.396	0.497
Ratio of CRABP2/FABP5 expression (≥1 vs <1)	0.746	0.388	0.615	0.735

Abbreviations: ESCC, esophageal squamous cell carcinoma; OS, overall survival; PFS, progression-free survival.

In Cox proportional multivariate analysis, death was defined as a dependent variable, and nine factors were defined as arguments such as the gender, ethnicity, differentiation of degree, depth of invasion, AJCC stage, lymph node metastasis, hematogenous metastasis, nerve invasion, CRABP2 expression, etc. Results of all the significant variables and patient survival are shown in Table 6. We found that nerve invasion (*β* = 0.522, HR: 1.685, 95% CI: 1.095–2.592, *p* = 0.018) was a risk factor for OS of ESCC (Table 6). In addition, compared with poorly differentiated, moderately differentiated (*β* = −0.381, HR: 0.683, 95% CI: 0.445–1.051, *p* = 0.083) and well differentiated (*β* = −0.977, HR: 0.376, 95% CI: 0.205–0.692, *p* = 0.002) were protective factors for the OS of ESCC. However, the *p*-value of CRABP2 was close to 0.05 and CRABP2 expression (*β* = −0.405, HR: 0.667, 95% CI: 0.440–1.011, *p* = 0.056) was probably protective factor for OS of ESCC in Table 6. Furthermore, recurrence was regarded as a dependent variable, and five factors such as differentiation of degree, AJCC stage, lymph node metastasis, hematogenous metastasis, and CRABP2 expression were regarded as arguments. The result of all the significant variables and patient survival are shown in Table 7. We found that compared with poorly differentiated, moderately differentiated (*β* = −0.525, HR: 0.592, 95% CI: 0.388–0.902, *p* = 0.015) and well differentiated (*β* = −0.958, HR: 0.383, 95% CI: 0.217–0.679, *p* = *p* = 0.001) were protective factors for the PFS of ESCC. Hematogenous metastasis (*β* = 0.800, HR: 2.225, 95% CI: 1.074–4.608, *p* = 0.031) was also found to be a risk factor affecting PFS. However, the invasion depth, AJCC stage, lymph node metastasis, and CRABP2 expression were not risk factors or protective factors for PFS of ESCC as shown in Table 7.

**Table 6:** Multivariate analysis of factors associated with OS for ESCC

	Reference group	*β*	*P*-value	HR	95.0% CI	
CRABP2		−0.405	0.056	0.667	0.440	1.011
Degree of differentiation	Poorly differentiated		0.007			
Moderately differentiated		−0.381	0.083	0.683	0.445	1.051
Well-differentiated		−0.977	0.002	0.376	0.205	0.692
Lymph node metastasis		0.473	0.272	1.604	0.690	3.733
AJCC		−0.112	0.811	0.894	0.357	2.239
Hematogenous metastasis		0.515	0.174	1.674	0.796	3.521
Ethnicity		0.055	0.766	1.057	0.735	1.518
Gender		−0.101	0.622	0.904	0.606	1.350
Invasion depth	Mucosa		0.925			
Muscularis		−0.113	0.777	0.893	0.409	1.953
Full thickness		−0.150	0.702	0.861	0.400	1.852
Nerve invasion		0.522	0.018	1.685	1.095	2.592

Abbreviations: CI, confidence interval; ESCC, esophageal squamous cell carcinoma; HR, hazard ratio; OS, overall survival.

**Table 7:** Multivariate analysis of factors associated with PFS for ESCC

	Reference group	*β*	*p*-value	Exp(B)	95.0% CI	
CRABP2		−0.174	0.389	0.840	0.566	1.248
Degree of differentiation	Poorly differentiated		0.003			
Moderately differentiated		−0.525	0.015	0.592	0.388	0.902
Well-differentiated		−0.958	0.001	0.383	0.217	0.679
Hematogenous metastasis		0.800	0.031	2.225	1.074	4.608
AJCC		−0.212	0.643	0.809	0.330	1.984
Lymph node metastasis		0.633	0.133	1.884	0.825	4.305

Abbreviations: CI, confidence interval; ESCC, esophageal squamous cell carcinoma; HR, hazard ratio; PFS, progression-free survival.

The Kaplan–Meier method was used to continuously analyze OS and PFS of ESCC patients with clinical parameters.

**Figure 7:**
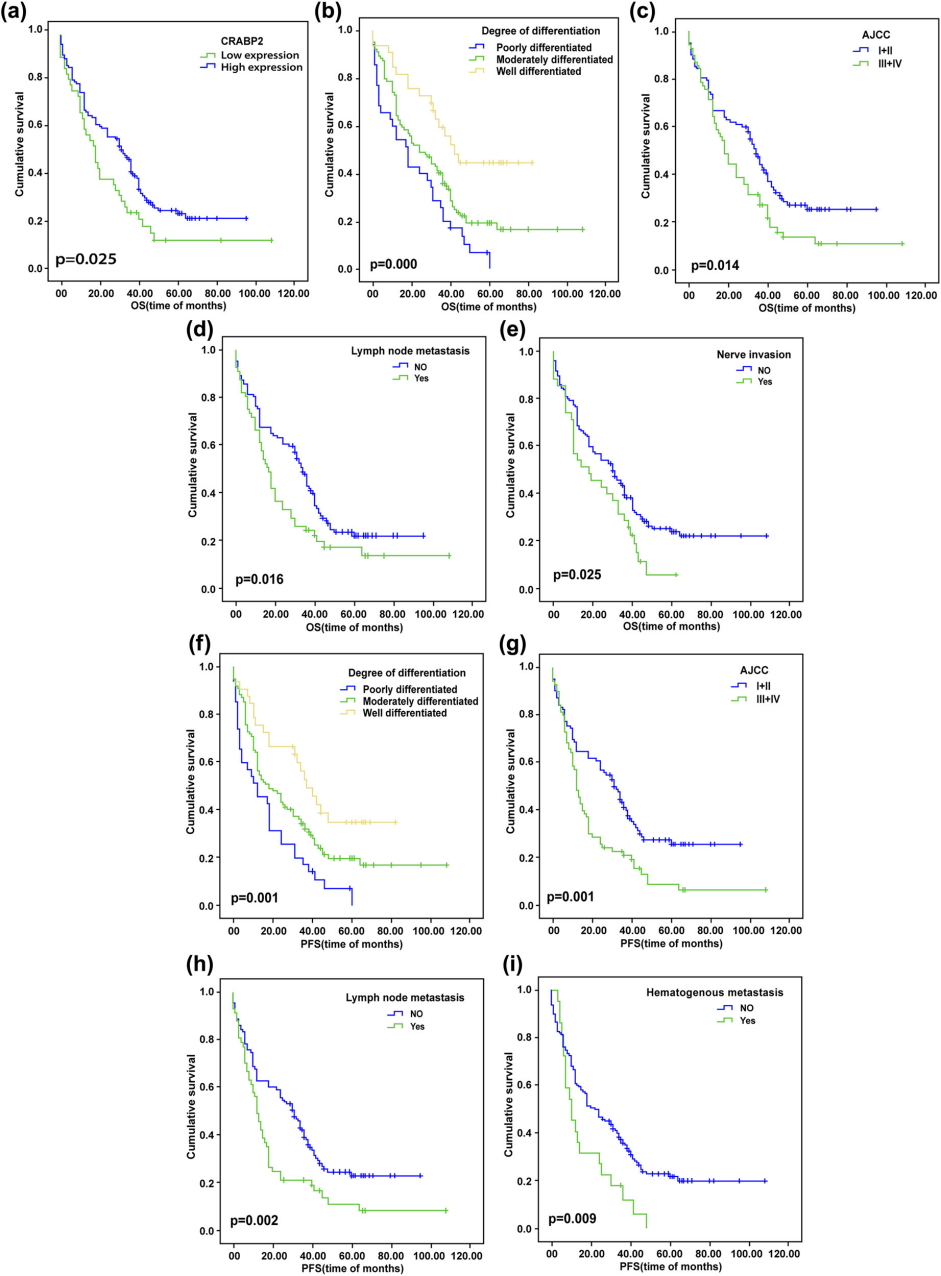
Kaplan–Meier survival curves for patients. (a) The overall survival of patients in ESCC with the expression of CRABP2; (b) the overall survival of patients in ESCC with a degree of differentiation; (c) the overall survival of patients in ESCC with AJCC; (d) the overall survival of patients in ESCC with lymph node metastatic; (e) the overall survival of patients in ESCC with nerve invasion; (f) progression-free survival of patients in ESCC with a degree of differentiation; (g) progression-free survival of patients in ESCC with AJCC; (h) progression-free survival of patients in ESCC with lymph node metastatic; and (i) progression-free survival of patients in ESCC hematogenous metastatic.

The results showed that the OS of patients with high expression of CRABP2 has a longer OS than the negative CRABP2 (*p* = 0.025) (Figure 7a). The OS of poorly differentiated patients was significantly shorter than that of patients with well-differentiated patients (*p* = 0.000) (Figure 7b). The OS of patients with early esophageal cancer (AJCC stage I and II) was better than that of patients with III and IV (*p* = 0.014) (Figure 7c). For patients with lymph node metastasis and nerve invasion, their OS time was also shorter (*p* = 0.016 and *p* = 0.025) (Figure 7d and e).

Besides, the PFS with poorly differentiated patients was significantly shorter than that of patients with well-differentiated patients (*p* = 0.001) (Figure 7f). The patients with AJCC stage I and II was longer than that in AJCC stage III and IV (*p* = 0.001) (Figure 7g). For patients with lymph node metastasis and hematogenous metastasis, their PFS time was also shorter (*p* = 0.002 and *p* = 0.009) (Figure 7h and i).

## 4 Discussion

ESCC remains a relatively poorly defined disease that lacks specific therapeutic targets and prognostic biomarkers. The molecular characterization of ESCC contributes to our understanding of its unique biology and could be translated into novel therapeutic strategies to improve clinical efficacy. With the rapid development of high-throughput sequencing technology and microarray hybridization technology, in order to meet the needs of researchers for information sharing, multiple gene databases have emerged and they have been widely used in gene expression quantitative analysis, providing a good platform for tumor research. The present study extracted TCGA and used the chip data in the library to explore the expression level of CRABP2 in esophageal cancer. In recent years, there were many studies on the abnormal expression of RNA and protein in tumors but the role of CRABP2 in esophageal squamous cancer is not fully understood.

CRABP2 gene is a member of the retinoic acid-binding protein family and lipocalcin/cytoplasmic fatty acid-binding protein family. Compared with other transport proteins in the family, CRABP2 has a higher affinity for RA [8], It preferentially binds to RA in the cytoplasm and transports it to the retinoic acid receptors (RARs) in the nucleus to activate the transcription of specific genes.

In breast cancer, lung cancer, liver cancer, and Wilms tumor, CRABP2 plays a role in promoting cancer [9,10,11,12,13]. CRABP2 regulates the HIPPO signaling pathway, large tumor suppressor (Lats1) stability promotes the invasion and metastasis of ER-breast cancer; in highly metastatic lung cancer cell lines, overexpression of CRABP2 can increase the level of RNA binding protein (HUR) and promote lung cancer cell invasion, migration, and anti-apoptosis through the transduction of integrin β1/FAK/ERK signaling pathway and the high expression of CRABP2 is associated with lymph node metastasis, poor overall survival, and increased recurrence. CRABP2 is highly expressed in poorly differentiated liver cancer, knocking down CRABP2 can inhibit the proliferation, migration, and invasion of liver cancer cell lines, promote cell apoptosis, and reduce the expression of ERK/VEGF pathway-related proteins. In Wilms tumor, the expression of CRABP2 is increased in samples of patients receiving preoperative chemotherapy and patients with metastatic disease. In non-small cells, the plasma CRABP2 concentration in lung cancer patients is significantly higher than that in the normal control group and is associated with a lower survival rate of non-small cell lung cancer. Testing the plasma CRABP2 concentration may be a new genetic testing method for the diagnosis of non-small cell lung cancer and the prognosis.

On the contrary, in malignant peripheral nerve sheath tumors (MPNSTs), head and neck squamous cell carcinoma, and esophageal squamous cell carcinoma, CRABP2 exert a tumor-inhibiting effect [14,15,16]. The high expression of CRABP2 may be a treatment for MPNST, and CRABP2 is not related to RA; CRABP2 has obvious nuclear staining in the normal squamous epithelium of the head and neck, but low expression in squamous cell carcinoma cells, and patients with high CRABP2 expression have longer overall survival. In the ESCC tissue, the expression of CRABP2 was significantly downregulated. Overexpression of CRABP2 inhibited the growth, metastasis, and apoptosis of tumor cells and was closely related to tumor location, TNM stage, invasion depth, and degree of differentiation but the specific mechanism is unclear. The results are consistent with the results of our study.

Among the 20 CRABP2 interacting proteins screened, based on database comparison and literature review, the role of the relationship between CRABP2 and FABP5 in ESCC has aroused the interest of the research group.

The correlation between CRABP2 and FABP5 has been found in many cancer types: the mRNA and protein levels of CRABP2 and FABP5 in bladder cancer tissues (BC) are significantly higher than those in adjacent nontumor tissues, suggesting that the expression of CRABP2 and FABP5 in BC tissues is upregulated and they may become new biomarkers of BC [17]. The expression of CRABP2 and FABP5 in fibroblasts or pancreatic stellate cells (PSC) activated in PDAC and other diseased pancreas such as CC and CP was significantly lower than that of the rest of fibroblasts. In malignant gliomas, the median ratio of FABP5/CRABP2 mRNA expression of 3.6 in short-term survivors (≤6 months) was significantly higher than that of long-term survivors (≥36 months) of 1.4, indicating that the FABP5/CRABP2 ratio can determine the direction of cell differentiation [18].

Some scholars disagree with the view that the ratio of FABP5/CRABP2 determines the direction of cell differentiation: Bai Gang believe that the ratio of CRABP2/FABP5 cannot be used as a target for retinoic acid to act on craniopharyngio cells, and the proportional relationship between RARα and PPARβ/δ may be the molecular target that determines the ultimate effect of retinoic acid [19]; in another glioblastoma study, it was found that the expression patterns of CRABP2 and FABP5 had nothing to do with the tumor grade or the sensitivity of RA [20]. 4-Amino-2-trifluoromethyl-phenyl complex acid (ATPR) is a new type of retinoic acid derivative that can inhibit the proliferation of cancer cells and induce differentiation. However, the significant inhibitory effect of ATPR on the proliferation, invasion, and migration of breast cancer cells may not be directly related to the ratio of CRABP2/FABP5 [21]. In summary, the proportional relationship between CRABP2 and FABP5 with tumor progression is still controversial. But our team deemed that it is related to tumor types and specific pathological types, and the expression level and ratio of CRABP2 and FABP5 are expected to serve as valuable biomarkers.

This study confirmed by immunohistochemistry the following: (1) CRABP2 and FABP5 are both expressed in the esophageal squamous cell carcinoma tissue and normal esophageal squamous epithelium, but the expression of normal esophageal squamous epithelium is significantly higher and stronger than that in the ESCC tissue. The difference was statistically significant (*p* < 0.001). (2) In our results, the expression of CRABP2 is not statistically significant with ethic, lymph node metastasis, AJCC and clinical parameters. This does not mean that the expression of CRABP2 has nothing to do with the progress of the ESCC and we suspect that this result may be related to the sample variability of this study. (3) The expression of FABP5 is related to lymph node metastasis (*p* = 0.032), invasion depth (*p* = 0.041), and AJCC (*p* = 0.013). What makes us feel contradictory and interesting is that FABP5 should be a tumor suppressor in ESCC like CRABP2, with high expression in normal tissues and low expression in tumor tissues. Its high expression in tumor tissues will inhibit the development of the tumor. However, the fact is just the opposite: the high expression of FABP5 in ESCC promotes lymph node metastasis, deeper invasion, and higher stage, which is opposite to CRABP2. In addition, FABP5 was highly expressed in prostate cancer, intrahepatic cholangiocarcinoma, colorectal cancer, cervical cancer, and other tumor tissues, while was low expressed in normal tissues. Its high expression promoted tumor growth and metastasis [22,23,24,25,26], but the same results were obtained in this experiment. We speculate that this contradictory result may be related to more pathways involved in the occurrence and development of ESCC and the receptor protein downstream of the retinoic acid signaling pathway [27]. It is possible that the RA signaling pathway is not the main pathway of FABP5 protein. (4) CRABP2/FABP5 is related to ethnicity (*p* = 0.001), nerve invasion (*p* = 0.031), and chemoradiotherapy (*p* = 0.038), and the low ratio of CRABP2 and FABP5 boosts differentiation of tumor, indicating that CRABP2/FABP5 affected the progress of ESCC. Considering that both are closely related to retinoic acid, CRABP2 and FABP5 are expected to be targets for predicting esophageal squamous cell carcinoma and clinical treatment. (5) Our results found that CRABP2 is related to OS and not to PFS. This result is not consistent with that of Liu et al. [28], but consistent with others [5,29,30,31,32], However, FABP5, the ratio of CRABP2 and FABP5 are also not associated with OS and PFS. We assume it is related to the type of tumor and the difference in its role in tumors but further study is needed to ascertain. In summary, by integrating public databases, it is found that CRABP2 is significantly low in EC and may participate in the development of esophageal cancer through related metabolism, which has a certain reference value for clinical treatment and judging the prognosis of esophageal cancer. CRABP2 may be an effective molecular marker for diagnosing and predicting the development of ESCC. The biological relationship between CRABP2 and FABP5 can be used as a target for the treatment of esophageal cancer remains to be further studied by this group.
